# Photovoltaic Cells and Scintillators Towards Carbon Footprint Reduction: Advantages and Challenges for Ecological Safety

**DOI:** 10.3390/ma17235909

**Published:** 2024-12-03

**Authors:** Agnieszka Iwan, Krzysztof A. Bogdanowicz, Robert Pich, Agnieszka Gonciarz, Witalis Pellowski, Jacek Miedziak, Wojciech Przybyl

**Affiliations:** 1 Faculty of Security and Safety Research, General Tadeusz Kosciuszko Military University of Land Forces, Czajkowskiego 109 Str., 51-147 Wroclaw, Poland; robert.pich@awl.edu.pl (R.P.); agnieszka.gonciarz@awl.edu.pl (A.G.); witalis.pellowski@awl.edu.pl (W.P.); jacek.miedziak@awl.edu.pl (J.M.); 2 Military Institute of Engineer Technology, 136 Obornicka Str, 50-961 Wroclaw, Poland; bogdanowicz@witi.wroc.pl (K.A.B.); przybyl@witi.wroc.pl (W.P.)

**Keywords:** photovoltaic cells, scintillators, ecological safety, health toxicity, recycling, carbon footprint reduction, recycling, ESG strategy

## Abstract

The main goal of this review paper is to show the advantages and challenges of photovoltaic cells/modules/panels and scintillators towards carbon footprint reduction for ecological safety. Briefly, the various types of solar-driven CO_2_ conversion processes are shown as a new concept of CO_2_ reduction. The health toxicity and environmental effects of scintillators, along with risks associated with use and disposal, are presented, taking into consideration inorganic and organic materials. Factors affecting the durability and lifespan of scintillators and the carbon footprint of solar cell production are analysed, considering CO_2_ emission. Moreover, the technology of recycling photovoltaic modules and scintillators, along with a SWOT analysis of scintillation material toxicity, is presented to find the best solutions for clean technology and ecological safety. Finally, we offer recommendations for the areas where the most significant reductions in CO_2_ emissions are expected to be implemented in the future of green energy in industry, including ESG strategies.

## 1. Introduction

The global average annual temperature has risen significantly since the industrial revolution, and the last decade (2010–2019) was the warmest since temperature measurements began. Of the 20 warmest years in measurement history, 19 were recorded after 2000. According to data from the Copernicus climate change monitoring program [1], 2023 was Europe’s second-warmest year, at 1.02–1.12 °C above average. Temperatures in Europe were above average for 11 months of the year, and September was the warmest on record. Winter and autumn were both the second warmest on record. The Earth’s average temperature is now 0.95–1.20 °C higher than at the end of the nineteenth century. If temperatures rise by more than 2 °C over pre-industrial levels, the risk of dangerous and potentially catastrophic environmental changes will increase dramatically. Therefore, the international community has agreed that global warming should be kept below 2 °C. The primary cause of the temperature rise is the increase in greenhouse gas (GHG) emissions, mainly carbon dioxide (CO_2_). A significant source of GHG emissions is fossil fuel-based electricity generation. Burning coal in coal-fired power plants produces nearly 0.9 kg of CO_2_eq per kWh [2].

It is well known that greenhouse gases are the primary cause of global warming. They have the property of blocking infrared light while letting in visible light. However, the energy from the sun to the earth is primarily visible light, while the energy from the world to space is mainly infrared. This means that while the amount of incoming energy remains the same, the amount of outgoing energy decreases, increasing the earth’s temperature. Global warming caused by greenhouse gases leads to severe issues such as flooding, heat waves, cold waves, reduced sunlight reflectance, and rising ocean temperatures. In 2008, the EU set a goal of reducing greenhouse gas emissions by 20% by 2020 compared to 1990 levels to combat climate change. By 2019, emissions had fallen by 24% and 31% by 2020, partly due to the coronavirus pandemic. The new targets were set in 2021. The European Parliament approved the EU’s climate law, which makes climate neutrality by 2050 legally binding in the EU and sets an interim target of a 55% reduction in emissions by 2030 [3].

Due to the current increase in ecological awareness, society has begun to analyse all used products not only in terms of how eco-friendly they are to produce, but also whether the resources can be renewed or recycled and, more importantly, what will happen with the product when the utility is no longer met; hence, their entire lifecycle. In recent years, this trend has included the technology of renewable energy production, not only the chemical and physical phenomena but also the materials used in their production. One of the most important factors is the toxicity of photovoltaic technologies, which varies depending on the generation of cells and affects three main categories: selection of materials, production, use, and end-of-life.

The first generation of photovoltaic technology includes thick crystalline films based on mono-, poly- and multi-crystalline silicon and III-V single junctions like gallium arsenide (GaAs). This technology is the most widely spread worldwide and has the largest market share. A typical efficiency value ranges around 10–24% and a life span of 14–25 years for silicon-based solar cells, and 28–30% efficiency and an 18-year life span for GaAs [4]. At the level of the materials used for the construction, the silicon solar cell is based mainly on silicon with the addition of silver and aluminium and, in some cases, with smaller amounts of copper or lead. The production of first-generation cells involves a high-temperature and energy-consuming process in which silicon wafers are made. Although silicon as a material is relatively neutral for the environment, toxic gases, like silicon tetrachloride, are generated during its processing. In terms of the use of silicon solar cells, they are relatively very stable during their operation and emit no toxic compounds to the environment. The recyclability when reaching the end-of-life stage should involve caution due to the presence of lead in solder, which would present a hazard to the environment due to the scale of its use in such cells [5].

The second generation of photovoltaic devices consists of the development of thin-layer materials such as microcrystalline silicon (μc-Si), amorphous silicon (a-Si), copper indium gallium selenide (CIGS), and cadmium telluride/sulphide (CdTe/CdS). The main drive for developing thin-layer technology was in reducing the amount of semiconductor materials used for production. The biggest challenge is scaling up, in terms of transporting the efficiency value from the laboratory setup, which ranges from 23.4% for a laboratory setup up to approx. 20% for a module. The expected lifespan ranges between 12 and 20 years, with the lowest life expectations for CIGS and the highest for CdTe/CdS cells [4]. For comparison’s sake, the materials used for producing second-generation solar cells apart from silicon involve cadmium, tellurium, copper, indium, gallium, and selenium. The production process is based on co-evaporation, evaporation, sputtering, and, in some cases, solution deposition techniques. However, control over the stoichiometry of the formed thin films is crucial to ensure the best quality of the absorber layer. These processes are also very demanding, as waste management needs to be taken into concern because of toxic and cancerogenic elements like cadmium and hydrogen selenide.

Moreover, these solar cells use rare materials such as indium and gallium, whose extraction dramatically impacts the environment. CIGS and CdTe/CdSe-based solar cells are relatively safe thanks to the applied encapsulation method. However, theoretically, in case of physical damage, the danger of contamination and intoxication of the environment is highly probable. Compared to silicon solar cells, the recyclability processes for CIGS and CdTe/CdSe-based solar cells are poorly developed. The CdTe solar cells must be carefully handled to prevent potential cadmium pollution [6].

The third generation of solar cells consists of several technologies, such as perovskite, dye-sensitized, and organic and tandem structures. This generation represents a group of devices with widely spread performance and lifespan values, ranging from 5 to 9% for dye-sensitized solar cells and organic solar cells up to above 20% for perovskite solar cells. Due to the high sensibility of the materials and technological limitations, the comparable life span for third-generation solar cells is difficult to assess sharply. Usually, it lasts months on a small laboratory scale [4]. When it comes to material selection for the construction of third-generation solar cells, the potential impact of many organic compounds of diverse molecular structures on human health or the environment is currently unknown. Some solar cell and module structures rely on volatile organic solvents or lead-based compounds. The production of this generation of solar cells needs to be considered highly hazardous due to the use of materials and processes that generate heavy by-products and recycling waste. Their biggest downside is their limited longevity caused by the degradation of the absorber. Third-generation solar cells suffer mainly from limited recyclability and low stability of the absorber material and require careful handling due to potential lead pollution [7].

**Figure 1:**
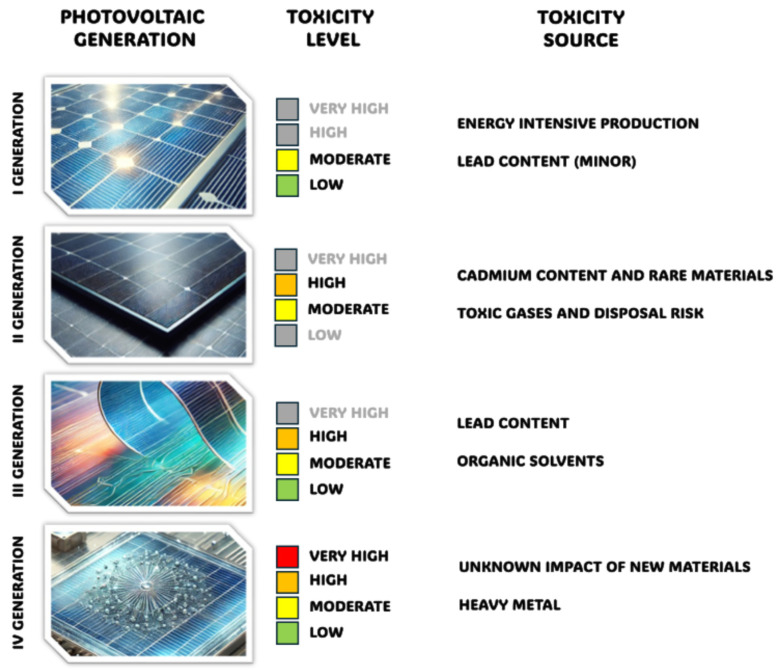
Graphical representation of toxicity and their main sources for all four solar cell generations [4,5,6,7].

The fourth generation, known as nano-photovoltaics, includes quantum dots, multijunction photovoltaics, and emerging technologies. In some publications, they are included in the third-generation solar cells; however, for clarity’s sake, we will refer to them as the fourth generation. The efficiency of quantum dot-based photovoltaics yields below 20%, whereas the multijunction ones reach almost 40% (mainly those which contain tandem architecture). Quantum dot technology is based on epitaxial nanocrystal growth and sometimes requires heavy metals like cadmium or lead. Other representatives of this group are largely in the experimental stage, using various materials and structures; therefore, the toxicity of materials varies considerably [7]. The assessment of longevity is even more difficult to assess due to the wide variety of materials and technologies. The samples are usually prepared on a laboratory scale with fractions of square centimetres in size. The life span expectation is similar to or lower than the previous generation [4]. Given the low technological readiness level, their toxicity and other aspects must be further studied. Figure 1 presents a general summary of solar cell generation linked to the assessed toxicity source and assigned toxicity level.

The projected number of PV panels that will be disposed of in 2050 is 9.57 million tonnes. Taking into account device structure and architecture, solar cells are classified as either wafer-based solar cells or thin-film technologies. The life cycle of solar cells starts from the extraction of raw materials and ends with the disposal or recycling and recovery of the PV components (after a lifespan of 25–30 years, in the case of silicon solar cells). Currently, life cycle assessment (LCA) of thin-film solar cell technologies has focused on two indicators: (i) climate change and global warming impact category (greenhouse gas emissions) and (ii) energy-related indicators such as cumulative energy demand (CED) and energy payback time (EPBT). At the same time, it should be noted that one or both indicators are discussed in scientific papers. Moreover, a life-cycle energy analysis accounts for both the input energy (required for production and maintenance of the system) and the output, or electrical energy generated by the system over a yearly cycle. It is important that the energy supplied by the system over its operational lifetime is significantly greater than its embodied energy. Additionally, the net emissions of greenhouse gases from the PV system over its life cycle should be significantly lower than the emissions from competing fossil fuel options. The life-cycle energy requirement for emerging thin-film technologies such as perovskite solar cells (PSC), PSC tandem, DSSC, organic solar cells, colloidal quantum dot solar cells, kesterites, or copper zinc tin sulphide solar cells ranges from 103 to 3546 MJ/m^2^. Moreover, the energy payback time varies from 0.43 to 7.12 years, while the global warming potential (GWP) is in the range of 5–286 KgCO_2_eq/m^2^. In comparison, the life cycle energy requirement of commercial thin-film solar cell technologies such as a-Si, CIGS, CIS, CdTe, GaAs, and GaAs tandem is in the range of 1054–7939 MJ/m^2^. For EPBT and GWP, these were 2.11–6.35 years and 61–437 KgCO_2_eq/m^2^. The observed differences in the above values are mainly due to the energy and materials used in production, which have been significantly improved for emerging thin-film technologies [8].

One critical approach to reducing GHG emissions is decarbonising the electricity sector through alternative technologies like photovoltaics (PVs). In 2021, about 28.7% of the world’s electricity was produced from renewable sources [9]. By 2050, 85% of the world’s electricity will be produced from renewable sources [10]. The growing demand for renewable energy sources has led to a significant increase in the production of solar panels around the world. The installed global capacity of photovoltaics (PVs) has increased from 40 GW in 2010 to 709 GW in 2020. The world finally reached 1 TW of cumulative installed PV capacity in 2022 and is expected to reach 8.5 TW in 2050 [10,11].

The carbon footprint of a photovoltaic installation is related solely to its production. Photovoltaic installations convert sunlight into electricity without emitting carbon dioxide. This fact makes it an environmentally friendly technology. When considering the carbon footprint of 30 years of operation of photovoltaic panels, it is associated with the following production steps:

- Quartz mining and metallurgical silicon production;
- Mining and production of aluminium;
- Obtaining crystals and manufacturing wafers;
- Production of other components necessary for module assembly (glass, cables, EVA layers, aluminium frames, etc.);
- Assembly of cells and modules;
- Assembly of PV systems (on the roof).

An additional aspect of the carbon footprint of PV panels may be their disposal method after a 30-year lifetime. Most PV panel components are recyclable, lowering the potential future carbon footprint.

The carbon footprint is the total amount of greenhouse gases (GHGs) emitted directly or indirectly during a product’s life cycle, usually measured in tons of carbon dioxide equivalent (tCO_2_eq). It encompasses all stages of a product’s existence, from raw material extraction to production, use, and disposal.

When evaluating the total carbon footprint of photovoltaic panels, the following criteria should also be considered:

- Where and how the metallic silicon and aluminium are produced;
- The type of silicon crystal: monocrystalline or multi-crystalline;
- The place and method of manufacturing the modules;
- The energy mix used;
- Panel construction: with aluminium frames or frameless glass–glass modules;
- Disposal method.

Moreover, carbon dioxide from fossil fuels, the primary energy source, accounts for about 76% of the total volume of greenhouse gases. Therefore, there is a pressing need for technologies that can reduce carbon dioxide while utilising renewable energy sources to replace fossil fuels. For example, fuelling technologies convert captured carbon dioxide into fuel substances, including carbon monoxide, ethanol, methanol, ethylene, and a variety of chemical raw materials. Moreover, photocatalysis is a crucial technology for achieving carbon neutrality and mitigating global warming, as it enables the reduction of carbon dioxide emissions.

The possibility of generating electricity from solar radiation is a well-known phenomenon and is used in the 21st century worldwide. However, the variability of weather conditions and global warming are the reasons for searching for other natural energy sources. For example, scintillators are materials that emit light by absorbing ionising radiation [12]. The operating principle of a scintillator under the influence of ionising radiation is based on the absorption of radiation energy by the material, leading to the ionisation and excitation of electrons which emit light when returning to the ground state. The emitted light is then converted into electrical signals that can be analysed regarding the intensity and energy of radiation. This theory integrates phenomena from quantum mechanics, optics, and radiation physics, allowing for effective detection and analysis of ionising radiation. Based on the number and energy of the recorded photons (light pulses), the energy of the primary radiation can be reconstructed. This process is based on statistical models of the interaction of radiation with the material and the amount of emitted light. According to the principle of energy conservation, the radiation energy is directly proportional to the intensity of the emitted light. The energy of the emitted photon E_photon_ is related to the energy difference between the energy states of the electron in the scintillation material:

E_photon_ = hν

where: h—Planck’s constant, ν—frequency of emitted light.

The efficiency of the scintillator (how much light is emitted per unit of radiant energy) determines the luminescence coefficient L:

$$L=\frac{E_{light}}{E_{radiation}}$$

where: E_light_—energy of emitted light, E_radiation_—energy of radiation

**Figure 2:**
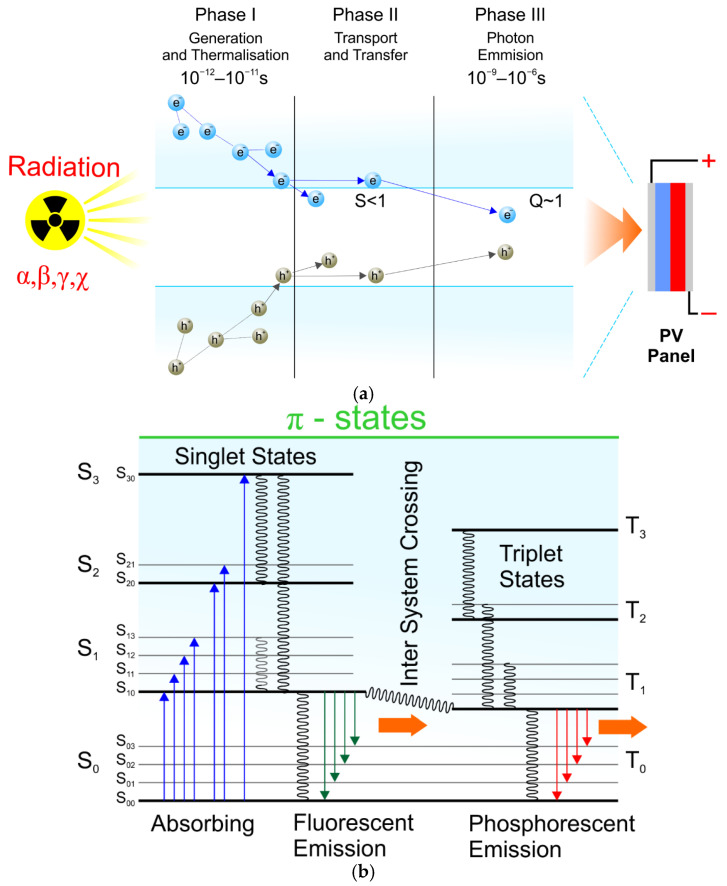
(**a**) The idea of converting radiation into photons of light. (**b**) There are three or two phases: the three-phase context involves generation and thermalisation (10^−12^–10^−11^ s), transport and movement of charges, and photon emission (10^−9^–10^−6^ s); the two-phase context involves generation and thermalisation and photon emission. The figure is an elaboration by this study’s authors, based on [13].

Scintillation is a quantum process described by the probability distribution of its occurrence in time. If a charged particle (α and/or β) or an energy photon (ɣ and/or X) passing through a scintillation material, it excites a certain number of atoms. When the atoms pass to the ground state according to stochastic relaxation, they release several photons, which decrease exponentially. The time-dependent number of photons is one of the key parameters when choosing the scintillator type. Usually, this time is less than 10^−8^ s, so detectors must be relatively fast. The scintillation material must have at least three energy levels in its structure: the ground, excited, and the third intermediate, which must be close to the excitation level. Then, the relaxation process takes place in two stages (according to the Jablonski scheme): first to the intermediate level, without light emission, and then to the ground state, with the actual emission of a photon in the form of light. Therefore, the existence of the third level is crucial. All emitted photons would be immediately reabsorbed in a configuration with only two levels; the scintillator would be opaque to its radiation. The idea of converting radiation into photons of light is presented in Figure 2.

Scintillators can be divided into categories depending on their chemical composition and properties. Each type has unique properties that determine its use in specific fields, such as nuclear physics, medical diagnostics, or radiation protection. Scintillators can be divided into several main categories: inorganic, organic, gaseous, nanocrystalline, and liquid. The selection of the appropriate scintillator depends on the application requirements, such as the type of radiation to be detected, light output, speed of response, and the cost and availability of the material. In addition, there are more advanced types, such as liquid and nano-scintillators (based on nanomaterials). Each type has subtypes and specialised applications [14,15,16]. Dopant elements such as thallium, cerium, europium, samarium, praseodymium, terbium, and lutetium are vital for improving the properties of scintillators, including their light output, decay time, and sensitivity to different types of radiation. These dopants enable scintillators to be used in various fields, from nuclear medicine to particle physics to radiation detection. Table 1 shows the most commonly used scintillator dopants and the functions they perform [17,18,19,20,21,22,23,24,25,26,27,28,29,30,31].

Inorganic scintillators [32] are made of high-density crystals and ceramics, which makes them ideal for detecting high-energy X-rays and gamma rays. The most commonly used scintillators contain sodium iodide doped with thallium (NaI(Tl)) or caesium iodide doped with thallium (CsI(Tl)) [33] and lead tungstenate (PbWO_4_) [17].

To achieve the ability to generate electrical energy from solar radiation via scintillators, independent of external factors (i.e., sunlight or another source of light photons), five suggested postulates must be met:

1. The structure of the scintillation layer should contain (structural) inclusions in the form of (for example) microcapsules of a radioactive substance. This will increase the spherical luminescence efficiency and improve the photons’ light transmission coefficient to the iso-solar cell’s surface (i-PV). The outer scintillation layer should be surrounded by a mirror material reflecting light towards the photovoltaic panel.
2. The possibility of both modulation of scintillation colours and observation with the naked eye is possible thanks to the doping of the materials with various elements. Initial observations of increased scintillation intensity and colour during X-ray exposure were observed after doping the organic structure with UO_2_^2+^. An increase in luminescence in the X-ray beam (E > 20 keV) was observed. Increased radiation resistance and reduced hygroscopicity were also observed compared to commercially available CsI:Tl or NaI:Tl scintillators [34].
3. A nuclear battery’s efficiency depends largely on matching the emitter (α, β, or ɣ source) to the scintillation material when converting radiation energy into photons of light. Nuclear batteries that use a single planar (layered) arrangement of components generally have low energy conversion efficiency. Spatial arrangements give much better results.
4. According to Dujardin [35], the scintillator material’s high natural radioactivity makes dopant contents of K, Rb, and Lu highly desirable in applications related to radiation conversion.
5. The perovskite single crystal can be a crucial scintillator in photovoltaic technology applications. It is characterised by high photoluminescence quantum efficiency and light efficiency conversion in a wide temperature range. According to literature reports [36,37,38], the emission lifetime is ~3.4 ns and the light efficiency is 1.5 ÷ 3 × 10^5^ photons/MeV. It may be proportional to the energy gap of these materials, which is estimated to be below 2 eV. The latest technology, quantum dots and nanocrystals, can also improve light conversion in perovskite scintillators. Pellowski et al. [39] demonstrated that converting ionising radiation into photons of light generated in the scintillator structure can be—if the appropriate material is selected—a sufficient light source to generate an electric charge in photovoltaic cells. A new approach to obtaining a source of photons based on the volumetric arrangement of a radioactive isotope in the scintillator structure presents rational premises for increasing light intensity. The layered arrangement of the module “responsible” for the light source described in the literature so far has been ineffective, and the efficiency of isotope cells is low. The concept of a new type of iso-photovoltaic cell (i-PV) proposed in the article may constitute a breakthrough in unconventional energy. Zero-emission and practically unlimited lifetime of such energy sources may significantly supplement the global energy mix.

Moreover, it should be stressed that among other alternative energy technologies that can harvest energies available in the ambient environment, mechanical energy can be captured and converted into useful electric power. Various energy-harvesting strategies have been proposed, including using electromagnetic, electrostatic, piezoelectric, triboelectric, thermoelectric, and pyroelectric transduction mechanisms at the meso-, micro-, and nanoscale, along with reducing the size of the device and making it flexible and mechanically resistant to damage [40,41,42,43].

It is known that the thermoelectric effect is based on the direct energy conversion between heat and electricity (Seebeck effect), or inversely, from an electrical current into heat (Peltier effect) without moving mechanical parts. Further research should be conducted on various thermoelectric materials and devices as promising alternatives to meet the challenges of the global energy impasse. For example, in [44] the authors investigated carrier and phonon transport control by domain engineering for high-performance transparent thin-film thermoelectric generators based on epitaxial SnO_2_ films with low thermal conductivity and high carrier mobility towards developing Internet of Things sensors.

Piezoelectric energy harvesting generally involves the conversion of mechanical energy (vibration, strain, or pressure) into electrical energy. This is based on the capacity of some materials to create an electrical potential difference when subjected to mechanical stress. For example, a transparent and flexible piezoelectric sensor for detecting human movement with a boron nitride nanosheet was investigated in [45].

The efficiency of energy-harvesting technologies is determined by the type of energy source and technology employed. Research into new materials and technologies is necessary for their application in a variety of industries, including Internet of Things, wireless communication, remote sensing, and biomedical applications.

Important factors affecting the efficiency of PV systems based on the conversion of solar radiation energy into electricity are geographical location, cloud cover, temperature, precipitation, and total exposure of the panel or place to light. Research is currently underway to create so-called all-weather cells, which are devices capable of generating energy from both the sun and rain by including an additional layer on the top surface of the cell. These are: piezoelectric devices; triboelectric devices, or storage of photoelectrons in phosphors with a long-lasting afterglow. In this type of piezoelectric device, the goal is to use the movement of objects such as raindrops (impact, splashing, bouncing, and spreading) to convert mechanical energy into electrical energy. The device consists of at least three layers, namely two electrodes separated by an inorganic piezoelectric material, e.g., lead zirconium titanate, barium titanate, or an organic material, as in the case of polymer ferroelectrics. This arrangement causes the polarization of +/− charges between the electrodes and the piezoelectric layer. The advantage is primarily the simplicity of execution. In addition, the thickness of the layers used is in the order of micrometres, thanks to which the devices are light and do not increase the weight of the entire system. The disadvantage of this solution is the amount of energy obtained, which depends mainly on the size of the droplet, the intensity of the precipitation, and the possibility of device deactivation as a result of covering the electrode with a thin layer of water. The best obtained result was an energy acquisition equal to 213 μW cm^−2^ (2076 μJ) at 7.6 V [46,47,48,49].

Another technology capable of obtaining electricity from rain is presented by triboelectric devices, which are made of a substrate on which an electrode covered with a hydrophobic layer is applied. The mechanism of energy generation is based on the transfer of electrostatic charge through a droplet moving on a hydrophobic surface. When the droplet comes into contact with the hydrophobic layer, the layer becomes polarized, and it moves with the droplet. The advantage is the use of transparent materials that can be applied to almost any surface. The disadvantage is the presence of ions in rainwater, as they generate an electrostatic charge. The best obtained result was a current value of approx. 270.0 μA at up to 143.5 V and approx. 50.1 W m^−2^ [50,51,52].

There have also been attempts to produce triboelectric devices with a silicon solar cell or dye-sensitized solar cells (DSSC). A poly(3,4-ethylenedioxythiophene) polystyrene sulfonate (PEDOT:PSS) electrode was inserted into an silicon heterojunction solar cell integrated into a triboelectric device. PEDOT:PSS was used to increase the short-circuit current density. Printed polydimethylsiloxane (PDMS) was used as a triboelectric material. The performance of the device was greatly improved by including a larger contact area between the raindrop and the printed PDMS. A current value of approx. 24.2 nA at a voltage of up to 3 V and a power of approx. 1.74 mW m^−2^ were obtained. However, further modification is required. [53]. In DSSC solar cells, graphene was applied on the back side of a polyethylene terephthalate (PET) substrate with an indium tin oxide (ITO) layer. A modified dye-based solar cell was built on the ITO layer, creating the following architecture: graphene/PET/ITO/DSSC. Due to the salt content of raindrops, the droplets fall on the surface of graphene and reach the periphery. A double-layer pseudocapacitor (EDL) is formed at the junction of the raindrop and graphene. The shrinking droplet releases electrons into the graphene, thus charging the pseudocapacitor. The brittleness of the glass resulted in the use of ITO instead of fluorine-doped tin oxide (FTO) in DSSC. A current value of 0.49 mA, a voltage of 109.26 mV, and a power of 54.19 pW (rainfall 20 mLh^−1^) were found [54].

One vision of the future is to obtain mechanical energy, especially taking into account the façade of the building, where energy would be generated not only by falling raindrops but also by vibrations caused by the wind. Depending on the architecture of the device, the angle of inclination, and the chemical composition of the raindrop, triboelectric systems could give values of 1 μWcm^−2^. Comparing the mechanical or electrostatic energy obtained from rain, it can be seen that the mechanical force would give a higher yield than the electrostatic forces.

To the authors’ best knowledge, there have not been any published works that compile information on solar cells and scintillators not only during use but especially during the production of individual devices. Therefore, the main goal of this review paper is to show the advantages and challenges of photovoltaic cells/modules/panels and scintillators towards carbon footprint reduction for ecological safety, considering the materials used. The organisation of this paper is as follows. Section 1 presents the toxicity to human health, environmental effects, and risks associated with the use and disposal of scintillators, considering inorganic and organic materials. In the subsections of Section 1, scintillators such as thallium-doped sodium iodide, thallium-doped caesium iodide, lead tungstate, and organic scintillators based on polystyrene, polyvinyl toluene, and anthracene, together with nano-scintillators, are described. Section 2 describes factors affecting the durability and lifespan of scintillators. In Section 3, the various types of solar-driven CO_2_ conversion processes are presented. Section 4 analyses the carbon footprint of solar cell production, considering CO_2_ emission. In the subsections of Section 4, silicon and aluminium production, together with mono and multi-crystalline silicon production for photovoltaic applications, are presented. Section 5 describes the technology of recycling photovoltaic modules, along with an analysis of the energy and economics of PV recycling. In Section 6, a SWOT analysis of scintillation material toxicity is presented, along with the recycling of scintillators and the impact of scintillators on CO_2_ emissions to find the best solutions for clean technology and ecological safety. Finally, we present recommendations for the areas where the most significant reductions in CO_2_ emissions are expected to be implemented in the future of the green energy industry, including environmental, social, and governance (ESG) strategies.

## 2. Results and Discussion

### 2.1. Toxicity and Effects of Scintillators on the Environment, Together with Risks Associated with Use and Disposal

#### 2.1.1. Thallium-Doped Sodium Iodide

Thallium-doped sodium iodide (NaI(Tl)) is one of the most commonly used scintillation materials in detecting radiation such as gamma radiation. This material’s toxicity and harmfulness depends on its components’ properties—sodium iodide (NaI) and thallium (Tl), doped in small amounts. Sodium iodide (NaI) is not considered highly toxic, although it can cause some skin, eye, and respiratory irritation in the form of dust or aerosol. When absorbed into the body in large amounts, sodium iodide can affect the thyroid gland, as iodine is one of the critical elements regulating its function. However, exposure to NaI does not pose a significant health risk under normal scintillator use conditions.

On the other hand, the main toxic hazard in NaI(Tl) comes from the admixture of thallium, which is highly toxic even in small amounts. Thallium can be neurotoxic, damaging the kidneys, liver, nervous system, and heart. Its compounds can penetrate the skin, increasing the risk of poisoning with prolonged contact. Although thallium is present in small amounts (0.1–0.2%) in NaI(Tl), exposure to its compounds can pose a severe risk, significantly if the crystals are damaged or the material is manufactured or processed incorrectly.

Taking into consideration the effects of NaI(Tl) on the environment, we would like to note that sodium iodide (NaI) does not pose a significant environmental risk. However, iodine in high concentrations can affect living organisms in aquatic ecosystems. From a pollution perspective, NaI is relatively stable and is not considered a severe environmental hazard under normal conditions. Thallium in NaI(Tl) is more problematic for the environment. Thallium and its compounds are toxic to plants and aquatic organisms and can accumulate in soil and water. Thallium is an element that migrates easily into the environment, which can lead to long-term damage to ecosystems. Its compounds can enter groundwater, pollute the environment, and cause serious ecological effects. Regularly using NaI(Tl) in scintillators (e.g., in radiation detectors) does not pose a significant hazard because thallium is firmly bound in the crystal structure, and the material is usually hermetically sealed. Toxic exposure may occur if the crystal is damaged (e.g., cracked or destroyed), which could release thallium dust. Inhalation of dust or skin contact may be harmful when disposing of or recycling NaI(Tl); special precautions should be taken to avoid the release of thallium into the environment. Waste containing thallium should be handled with attention paid to the regulations on hazardous substances. NaI(Tl) should be stored and used under conditions that minimise the risk of material damage and contact with dust. When working with NaI(Tl) in fine crystals or powder, appropriate personal protection (masks, gloves) should be used to prevent contact with thallium. Waste and damaged detectors containing NaI(Tl) should be disposed of safely and controlled. NaI(Tl) is not directly toxic under normal conditions of use, but the thallium it contains is highly poisonous to health and the environment. Thallium is particularly hazardous in the form of dust in the case of skin contact, as well as in aquatic and soil ecosystems. Appropriate protection against damage to the material and strict procedures for disposal are necessary to avoid potential hazards resulting from the presence of thallium.

#### 2.1.2. Thallium-Doped Caesium Iodide

Similar to NaI(Tl), CsI(Tl) is not considered highly toxic to humans under normal conditions of use; due to its thallium content, it may pose a potential hazard if exposed to dust, aerosols, or improper processing. The thallium in CsI(Tl) is highly toxic to humans and the environment, making this material potentially hazardous if improperly processed or disposed of. Caesium (Cs) as a solid element generally has low toxicity, although its radioactive isotopes (e.g., Cs-137) are hazardous. Caesium (Cs) itself is not considered a highly toxic substance, and its toxicity is more due to the risk of exposure to large amounts of dust or aerosol. It can cause eye, skin, and respiratory tract irritation if inhaled.

On the other hand, thallium oxide, even in minuscule amounts, is highly toxic to humans and animals. Thallium oxide is known for its neurotoxic effects and can also cause damage to the liver, kidneys, nervous system, and cardiovascular system. It is one of the most dangerous elements in terms of toxicity. Thallium can also easily penetrate the skin, which increases the risk of poisoning. In the CsI(Tl) scintillator, thallium is present in minimal amounts as a dopant (in the order of 0.1–0.5%), significantly reducing its direct toxicity risk during regular use, for example, in radiation detectors. Nevertheless, during production, processing, or damage to the material, there is a possibility of exposure to toxic thallium compounds.

Taking into consideration the effects of CsI(Tl) on the environment, we would like to stress that caesium iodide (CsI) itself is not considered to be particularly harmful to the environment. However, its release in large quantities into water or soil can lead to local pollution. Thallium is much more problematic from an environmental point of view. Thallium and its compounds are highly toxic to aquatic organisms, plants, and animals. Thallium compounds can accumulate in ecosystems, causing long-term damage, and can also contaminate groundwater and soil. Therefore, CsI(Tl) can be harmful to the environment mainly due to the presence of thallium, although low concentrations of thallium in the doped scintillator reduce this risk. However, improper handling of waste containing thallium, such as incineration or uncontrolled dumping, can lead to the release of toxic thallium compounds into the environment. For these reasons, during the production, use, and disposal of CsI(Tl), precautions are recommended to avoid inhalation of dust and skin contact and to prevent the release of thallium compounds into the environment. Waste containing CsI(Tl) should be disposed of following the regulations for hazardous materials due to the potential hazard resulting from the presence of thallium.

#### 2.1.3. Lead Tungstate

Lead tungstenate (PbWO_4_) is considered relatively safe under normal conditions of use because the toxic elements are chemically bound in a stable structure. However, the lead in this compound is toxic, especially in forms that the body can easily absorb. Therefore, care must be taken when processing, disposing of, or recycling this material to avoid the potential release of toxic substances. Its toxicity is determined by its components—lead (Pb) and tungsten (W)—which have different properties that affect its overall toxicity rating. Lead (Pb) is well known for its toxicity. Long-term exposure to lead, especially in its soluble form, can lead to several health problems, including damage to the nervous system, kidneys, and circulatory system. Lead is also toxic to the environment, and its compounds can contaminate soil and water. In the case of PbWO_4_, the lead is chemically bound in the crystal structure, which reduces its availability and potential toxic effects under normal conditions of use. The risk increases, however, when the material degrades, for example, due to mechanical or chemical damage, which can lead to the release of lead ions. Under normal conditions of use (in laboratories, radiation detectors), PbWO₄ does not pose a direct risk because lead and tungsten are tightly bound in the crystal structure, which limits their release. A potential risk occurs in situations where the material is subjected to high temperatures, acids, or mechanical damage, which could lead to the release of toxic lead and tungsten compounds into the environment or the body.

#### 2.1.4. Organic Scintillators Based on Polystyrene

**Figure 3:**
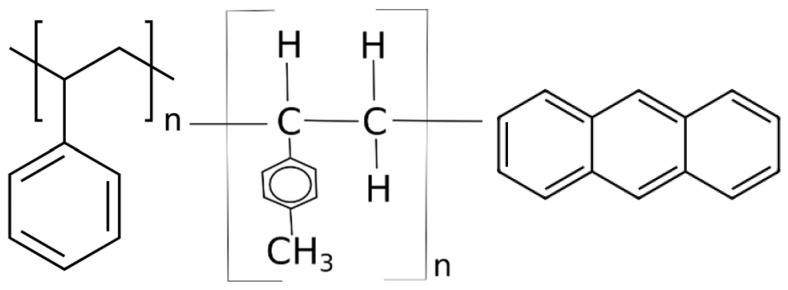
Chemical structure of polystyrene, polyvinyl toluene, and anthracene.

Unlike inorganic scintillators, organic scintillators (organic compounds that emit light when excited by radiation) have a lower density but a fast response time. As shown in Figure 3, common organic scintillators are polystyrene (PS), polyvinyl toluene (PVT) [55], and anthracene [56,57].

Polystyrene (PS) and polyvinyl toluene (PVT) are plastics commonly used in various fields, including as scintillation materials in radiation detection. Their toxicity and environmental impact depend on the chemical properties of these polymers and how they are used and disposed of. Solid polystyrene itself is not considered particularly toxic to humans. It is safe under normal conditions of use (e.g., in contact with food or detectors). Toxic risks may occur when polystyrene is burned or decomposed at high temperatures, releasing poisonous substances such as styrene, benzene, and other volatile organic compounds. Styrene (a monomer used to produce polystyrene) is considered an irritant and potentially carcinogenic to humans, especially in large quantities or with long-term exposure. The health risk of regular use of solid polystyrene is small, but it is advisable to avoid prolonged contact with fumes released when it melts or burns.

#### 2.1.5. Organic Scintillators Based on Polyvinyl Toluene

Polyvinyl toluene (PVT) is a polymer very similar to polystyrene, except that it contains additional vinyl and toluene groups. It is used as a scintillation material because it is characterised by greater mechanical strength and better optical properties than polystyrene. The toxicity of PVT in solid form is low, similar to polystyrene. Still, its combustion or thermal decomposition products can be problematic, resulting in toxic fumes, such as toluene and other volatile organic compounds, which can be harmful to health with long-term exposure. Toluene irritates the respiratory tract and central nervous system and, at high concentrations, can damage the liver, kidneys, and nervous system. Both polystyrene (PS) and PVT are difficult to biodegrade, making their disposal an environmental challenge. Polystyrene decomposes very slowly in the environment, contributing to pollution problems in oceans and other ecosystems. It is often found in the form of small particles that can be ingested by marine animals, leading to the accumulation of plastic in the food chain. PVT, like polystyrene, does not readily decompose in the environment. Its chemical durability means that PVT waste can remain in ecosystems for a long time, contributing to long-term plastic waste problems. Both of these polymers can also release microplastics into the environment, which is an additional environmental problem, especially in the context of water and soil pollution. Recycling polystyrene is possible, but it is often a costly and complicated process because polystyrene has a low density, which makes it difficult to transport and process. PVT is less frequently recycled due to specific applications (e.g., in scintillators), and its disposal often involves incineration in controlled conditions, which requires appropriate technologies to neutralise toxic emissions. Polystyrene and PVT have low toxicity in everyday use. Still, their combustion or thermal decomposition can lead to the release of poisonous substances such as styrene, toluene, and benzene, which are harmful to health. Both polymers are difficult to biodegrade, making them an environmental problem, especially in the context of plastic and microplastic pollution. Uncontrolled burning of these materials should be avoided, and appropriate recycling and disposal methods should be used to minimise their impact on health and the environment.

#### 2.1.6. Organic Scintillators Based on Anthracene

Anthracene is not considered to be highly toxic to human health at low concentrations. However, it can irritate the skin, eyes, and respiratory system, especially in the form of dust or in contact with fumes. Long-term exposure to large amounts of anthracene can cause skin irritation and ulceration. Anthracene itself is not classified as a carcinogen by the International Agency for Research on Cancer (IARC). However, many polycyclic aromatic hydrocarbons (PAHs), of which anthracene is one, have been associated with potential carcinogenic effects, especially after long-term exposure to PAH mixtures (e.g., during work in conditions of exposure to exhaust fumes, cigarette smoke, etc.). Anthracene enters the body primarily through the respiratory system and through the skin, but its systemic toxicity is low compared to other PAHs, such as benzo[α]pyrene. Anthracene is a toxic substance to aquatic organisms. High concentrations of anthracene in water can be harmful to fish, invertebrates, and aquatic plants. Anthracene, like other PAHs, can accumulate in organisms, leading to biomagnification—a phenomenon in which the concentration of toxic substances increases at higher levels of the food chain.

Moreover, anthracene is poorly soluble in water and relatively persistent in the environment. Its biodegradation in soil and water is slow, leading to its long-term presence in polluted ecosystems. Anthracene can undergo photodegradation under the influence of sunlight, which accelerates its decomposition, but this process is significantly slowed down in dark conditions. Anthracene itself has moderate toxicity to human health, but its irritating effects and potential accumulation in the body with long-term exposure may pose a risk. Anthracene is toxic to aquatic organisms, and its presence in water and soil may lead to long-term ecological problems due to its persistence and tendency to accumulate in the environment. The emission of anthracene into the environment should be limited, and appropriate methods of disposal and control of its emission in industrial processes should be used.

#### 2.1.7. Nano-Scintillators

Nano-scintillators based on nanomaterials, such as quantum dots, offer a modern approach to radiation detection. Due to the small size of nanoparticles, they can have unique optical properties and better light emission efficiency [58]. Modern scintillators, such as perovskites and other nanocrystalline materials, are gaining popularity because of their unique optical and electronic properties. They offer greater control over light emission and can be more tailored to specific applications. Perovskite scintillators such as CsPbBr_3_—caesium lead bromide—provide high light efficiency and the ability to adjust the wavelength of emitted light.

## 3. Factors Affecting the Durability and Lifespan of Scintillators

The lifespan of scintillators is primarily influenced by factors directly affecting their radiation-related properties, such as moisture, temperature, material ageing, and mechanical characteristics. To increase their durability, scintillators must be stored appropriately, protected from moisture and UV radiation, and be subjected to as little mechanical damage and excessive thermal stress as possible. Table 2 shows the key factors that affect their durability and lifetime [14,17,59,60,61,62,63,64,65,66,67,68].

Analysis of the latest literature reports allows us to summarise the high application potential of scintillator devices for converting photons of light generated by radiatively induced photoluminescence into electrical energy in iso-photovoltaic (i-PV) cells or detectors. The following parameters should characterise the scintillation material for i-PV detectors:

- Have high light efficiency (high efficiency—use of ionising radiation energy);
- Scintillations with long luminescence times;
- The wavelength emitted is consistent with the maximum sensitivity of the photocell;
- Non-hygroscopicity;
- Must be transparent to its light—minimal losses due to self-absorption;
- Effective collection of reflected internal light (high refractive index).

Many authors [69,70,71,72] point to the increasingly widespread use of scintillators in various fields of science and technology. Among the best practices for handling scintillators, the following should be highlighted:

- Proper labelling: Scintillator materials should be labelled appropriately to inform operators of potential hazards;
- Personnel training: Personnel handling scintillators should be trained in radiation safety and proper disposal methods;
- Monitoring: Regular monitoring for radioactivity and contamination should be performed when handling scintillators.

## 4. Types of Solar-Driven CO_2_ Conversion

**Figure 4:**
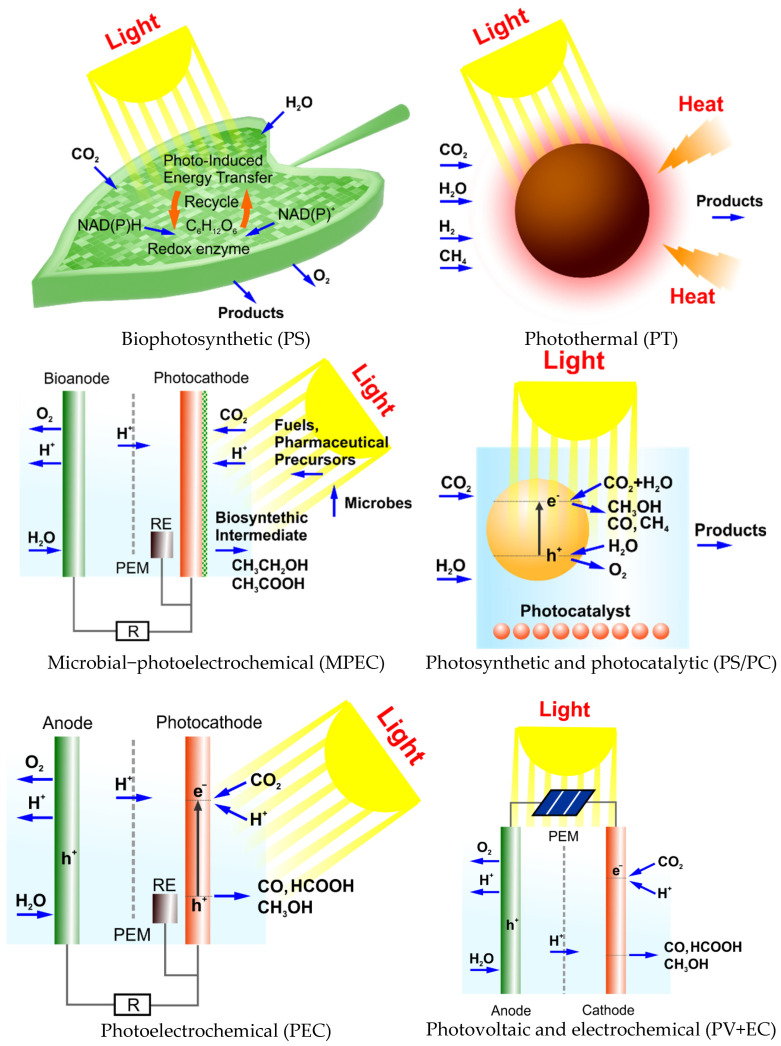
Schematic of types of solar-driven CO_2_ conversion. Own elaboration based on [73].

Photosynthetic (PS), photocatalytic (PC), photoelectrochemical (PEC), and photovoltaic with electrochemical (PV+EC) are classified as the various types of solar-driven CO_2_ conversion processes, as schematically presented in Figure 4.

Briefly, PS and PC use particulate or molecular photocatalysts, either in solution or immobilised on a surface. Both PC (ΔG < 0) and PS (ΔG > 0) processes depend on the oxidation half-reaction. In PEC technology, both (one) electrodes of the electrochemical cell are semiconductor photoelectrodes. Photogenerated charge carriers drive either one or both half-reactions. In PV+EC technology, a device is created via the combination of PV cells with CO_2_ electrolysis. This method decouples the light-harvesting and the electrochemical conversion steps. However, these are only laboratory studies at the moment; maybe in the future, the PV+EC technology approach will lead to industrial technologies [73]. Low solar-to-fuel efficiency, poor product selectivity, and unsatisfactory stability continue to impede the application of these technologies on an industrial scale. Highly efficient solar-driven CO_2_ reduction also requires solar cells and active scintillator materials for CO_2_ conversion (electrodes, catalysts) and a rationally planned device architecture. A higher CO_2_ conversion efficiency is possible when the device has enhanced light absorption, improved mass transfer, and optimal technological parameters (flow of gases and liquids) [74].

## 5. The Carbon Footprint of Solar Cell Production

With growing environmental concerns, the generation of electricity from solar energy is positioned today as a renewable “clean” energy. This positive effect explains the significant increase in the share of photovoltaic (PV) energy in the total amount of electricity generated over the past ten years. The manufacturing process is the only source of carbon emissions for a solar system because once installed, it is self-dependent. Therefore, it does not produce emissions, nor does it require an energy supply. Nevertheless, the manufacturing process is quite complex and necessitates energy expenditures.

### 5.1. Silicon and Aluminium Production

The primary raw material for PV modules is silica, a mineral that is very abundant on Earth. Silica production has increased globally from 2000 to 2019 by 240%. The transformation of silica to silicon of a purity suitable for producing high-performance silicon photovoltaic cells is a highly energy-intensive process. It is associated with CO and, in the next step, CO_2_ emissions. The carbon footprint of metallic silicon production can range from 4.7 kg CO_2_eq/kg Si to 16 kg CO_2_eq/kg Si, depending on the source of electricity used in the process, with the lowest footprint obtained if renewable or nuclear energy is used. In contrast, coal-only thermal energy leads to a triple carbon footprint [75].

Due to the photovoltaic industry’s high demand for aluminium, its production is also increasing, and with it, its carbon footprint.

From 2010 to 2018, global aluminium production grew by 52%. In 2018, the total world production of primary aluminium was 64.3 million metric tonnes, of which China contributed 57% [76]. The year 2019 presented a scenario that would see a need to increase the annual primary aluminium production over the next 20 years up to 90 million tonnes [77]. This means an increase of about 25 million tonnes from 2018 to 2040.

The problem with this is that primary aluminium is still primarily produced in smelters powered by coal-generated energy. In 2021, 67% of aluminium production came from fossil fuel power, mainly coal (57%) and natural gas (10%) [78].

In 2019, the average carbon footprint of aluminium production (for aluminium electrolysis) in different regions of the world was (in kg CO_2_eq/kg Al): in Europe—5, in North America—6.5, in South America—6, in Africa—11.2, in China—17.1, and in the rest of Asia—19.2. As of 2021, the total global average carbon footprint from aluminium production is 14.3 kg CO_2_eq/kg Al, and 70% is due to the production of the electric power used in the process [79].

So, we are creating material for devices that are supposed to protect us from global warming, while still contributing to CO_2_ emissions. Ideally, metal fabrication facilities would be based solely on renewable energy. The PCC BakkiSilicon hf. Plant, based in Iceland, produces silicon metal at one of the world’s most modern production facilities, using 100% energy from geothermal sources [80].

In addition to silicon and aluminium, other metals, such as copper, silver, indium, and tellurium, make up the composition of solar panels. Extraction of these metals produces carbon dioxide emissions. It is challenging to quantify emissions from mining such metals due to a lack of data transparency.

### 5.2. Production of Mono and Multi-Crystalline Silicon for Photovoltaic Applications

**Figure 5:**
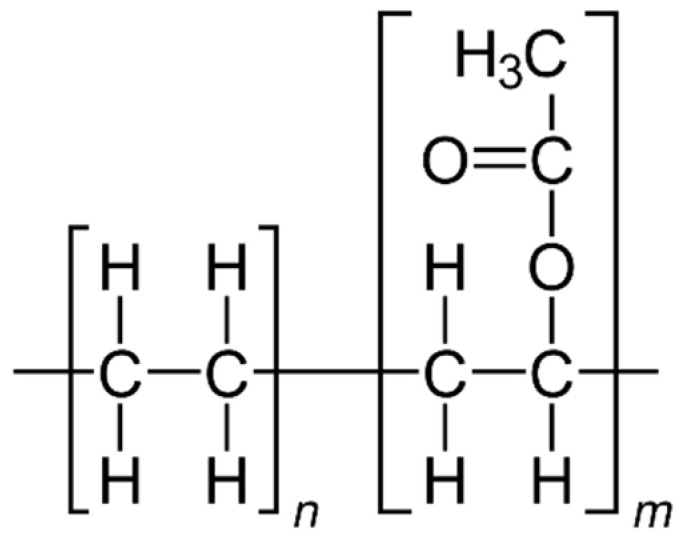
Chemical structure of EVA.

Among all PV technologies, monocrystalline (mono-Si) and multi-crystalline (multi-Si) silicon PVs are the most widely installed and have the highest global market share (95% in 2021). Regardless of the cell technology, silicon modules have a similar design. Silicon-based cells are sandwiched between encapsulant (ethylene vinyl acetate—EVA; see Figure 5) layers, with tempered glass as the top layer and a polymer-based backsheet. These different layers are usually enclosed in an aluminium frame [81].

Monocrystalline and multi-crystalline silicon PVs and thin-film solar PV panels require different manufacturing processes. Monocrystalline solar panels are the most efficient panels you can buy right now and are the most expensive, too. These panels are made from pure, single-cell silicon crystals cut from one big silicon slab. This process is complex, uses a lot of energy, and comes with the highest emissions of CO_2_ out of the three main types of solar panels. However, the high efficiency of monocrystalline solar panels means that they will recoup their emissions quicker than multi-crystalline panels. In addition, they also last longer; monocrystalline panels last up to 40 years, compared to the 25–30 years expected of multi-crystalline. They will therefore spend more time being carbon negative. Multi-crystalline panels are slightly less efficient than monocrystalline panels, and are also made differently. Instead of being made from a single slab of silicon, multi-crystalline panels use melted silicon crystals which are poured into a mould. This uses a large amount of energy, but still not as much as it takes to create monocrystalline panels.

There is also a significant environmental advantage to CIGS thin-film solar panel technology compared to crystalline silicon panels. Manufacturing Si PV modules produces an equivalent pollution of 50–60 g of CO_2_eq/kWh, while a CIGS solar panel only produces 12–20 g of CO_2_eq/kWh, which is similar to wind power that makes 10–12 g of CO_2_eq/kWh.

M. J. de Wild-Scholten calculated carbon footprints of 38.1, 27.2, 34.8, 22.8, 15.8, and 21.4 g CO_2_eq/kWh for monocrystalline silicon, multi-crystalline silicon, amorphous silicon, “micromorph” silicon, cadmium telluride, and CIGS rooftop photovoltaic systems, respectively. This calculation assumed a poly-silicon production with hydropower; ingot-, wafer-, solar cell, and module production with UCTE electricity; irradiation on an optimized angle of 1700 kWh/(m^2^• year); excluding installation, operation and maintenance, and the end-of-life phase [82].

### 5.3. PV Panel Production Location and Energy Mix Used

The carbon footprint for photovoltaic panels is heavily influenced by the energy mix of the country where the systems are produced. China currently has a dominant position in the market. In 2019, China produced 68% of polysilicon, 96% of all wafers, 76% of solar cells, and 71% of all PV modules. China accounts for half of the world’s PV panel production and produces twice as many CO_2_ emissions per panel compared to Western countries. This is because in China, energy is mainly generated from fossil fuels (69% of electricity from coal), and renewable energy is only a small part of the electricity mix. In contrast, the total energy supplied from renewable sources, such as in Germany or EU countries, is much higher.

**Figure 6:**
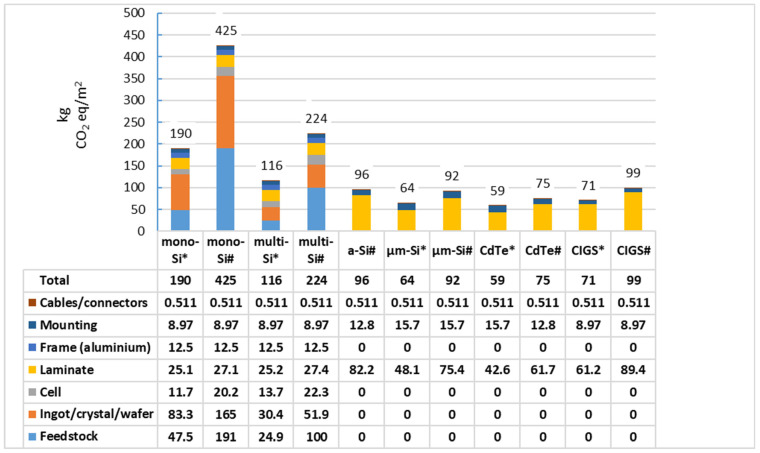
Carbon footprint for PV modules considering cell type and place of manufacture (EU/China). * modules produced and installed in the EU, # modules produced and installed in China. This figure is an elaboration by this study’s authors based on [82].

Furthermore, emissions are very different within varying Chinese provinces, being recorded in a range between 300 and 1050 gCO_2_eq/kWh. The average value at the national level is 750 gCO_2_eq/kWh. As a result, the fabrication of a 2 kWp PV installation in China generates 3.74 tons of CO_2_ [83]. Figure 6 compares the carbon footprint of various PV modules produced in different locations.

The authors of [82] provide carbon footprints for different types of photovoltaic cells. Conclusions from the comparison of carbon footprint values refer to cells of the same structure and technical solutions, but differing in the production process, especially the place of production and the related approach to environmental protection. Figure 6 shows data for five types of cells. Each cell type is represented by two cells: one manufactured in the EU and the other in China.

As can be seen in Figure 6, the carbon footprint clearly depends on the place of production. In factories, due to the lack of internal regulations limiting CO_2_ emissions, producers do not care about pro-environmental activities but only the company’s profit. Therefore, PV systems in China have a larger carbon footprint than those produced in Europe.

However, it should be stressed that factors affecting silicon solar cell efficiency include temperature (higher temperatures reduce efficiency), solar irradiance (greater sunlight intensity leads to higher efficiency), angle of incidence (optimal angles maximize efficiency), dust and dirt accumulation (reduces efficiency by blocking sunlight), and shading (partial shading decreases efficiency) [83].

Finally, it should be stressed that there are many papers devoted to the analysis of the impact of the type of material or solar cell architecture on photovoltaic parameters, although mainly for organic or perovskite cells, which also include the influence of electrode type, hole-transporting layer (HTL), electron-transporting layer (ETL), or active layer on the PV properties [84,85].

### 5.4. Modules with Glass Instead of Backfilm

**Figure 7:**
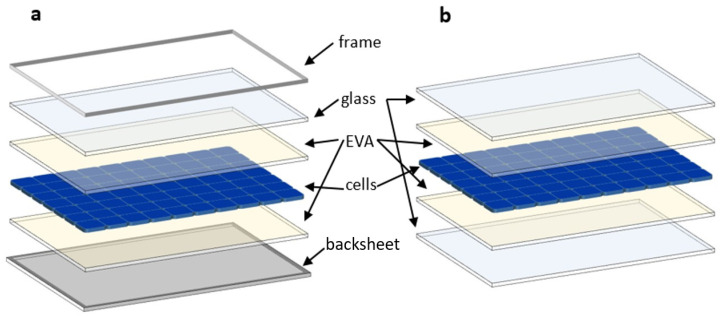
Comparison of the structure of glass–backsheet (G–B) module (**a**) and glass–glass (G–G) module (**b**).

The novel frameless glass–glass (G–G) modules are an alternative to the conventional backsheet (G–B) layout. The G–G design has emerged as a promising alternative, with a 10% market share in 2019 and an expected 30% market share by 2030 [86,87]. Figure 7 compares the structure of G–B and G–G modules.

Frameless glass–glass modules emit an average of 7.5 to 12.5% less CO_2_ than backsheet modules, regardless of where they are manufactured. This is because glass–glass modules do not require an aluminium frame, which is very energy intensive. In addition, glass–glass modules have a longer lifespan and lower annual degradation than film modules, further improving their carbon footprint.

Reichel et al. [88] compared CO_2_ emissions for two different solar module designs (conventional backsheet or novel frameless glass–glass modules) produced at three different locations (China, Germany, or the European Union). The study’s results show that CO_2_ emissions for backsheet modules are 810 kg of CO_2_eq/kWh in China, 580 kg in Germany, and 480 kg in the European Union. They are 750, 520, and 420 kg of CO_2_eq/kWh for glass–glass modules, respectively.

Therefore, glass–glass modules have a lower environmental impact than backsheet modules, and production in the EU and Germany has a lower environmental impact than production in China. As mentioned above, the main reason for this difference is the specific electricity mix at the production site.

## 6. Recycling of Photovoltaic Modules

Recycling photovoltaic (PV) modules is essential for economic and environmental reasons. Materials from PV components can be recycled using physical and chemical processes. However, a distinction must be made between recycling PV modules and recycling electronics, which are essential for the proper operation of the photovoltaic panel system.

PV component parts that have reached the end of their useful lives can generate toxic substances that potentially risk the environment and human health. The most harmful metals for health and environmental quality are cadmium, nickel, copper, lead, and silver. Recycling processes for these materials could reduce their negative impact on the environment.

Both recycling and reusing PV are better for the environment and economy than landfill or incineration. Recycling can reduce the need for new materials and the associated energy use and emissions, which is especially important given concerns about future potential material shortages. The percentage of PV waste in new installations is estimated to increase from 0.1% in 2016 to about 80% in 2050, indicating a growing demand for PV recycling solutions [89].

Photovoltaic (PV) technology has grown rapidly in recent years and has become a fundamental element of sustainable energy production worldwide. This growth is contributing to the development of technologies for recycling photovoltaic panels. The developed recycling process designs require adaptation to the structure and composition of photovoltaic modules and differences between technologies. Crystalline silicon and thin-film module technologies can be distinguished. Recycling crystalline silicon modules is a complex, multi-step process that aims to recover valuable materials and reduce environmental impact.

The recycling process generally begins with collecting and transporting used materials to specialised recycling facilities. The main goal of such recycling is to recover valuable materials such as glass, aluminium, and silicon. At the facility, sorting and pre-processing begin. This may include some or all of the following steps:

➢ Removal of frame, junction boxes, and cables;
➢ Separation of glass and silicon wafers;
➢ Separation and purification of silicon cells and metals.

Clean glass and metal frames can be reused for various applications. Silicon, after the specified cleaning process from plastic and metal layers, can be reused to produce new solar cells or used in silicon-based industries.

Recycling thin-film modules such as cadmium telluride (CdTe), copper, indium, gallium selenide (CIGS), and amorphous silicon (a-Si) requires specific methodologies adapted to each type. The aim of such recycling is to separate and recover valuable materials such as copper, indium, gallium, and selenium. The process starts with shredding the modules, thermal treatment to separate the materials, and undergoing a purification process to mitigate the environmental risk. The recovered CdTe can be used to re-manufacture the modules. It should be noted that thermal treatment plays a crucial role in the recycling. Different module compositions require specific thermal treatments adapted to the material to be recovered. For this purpose, heating, melting, or pyrolysis are used to separate and recover materials such as silicon, metals, and glass.

In the case of cadmium, which is a toxic element, the processes must be carefully carried out so that the risk to the environment is minimal. Cadmium poses one of the most serious threats to the natural environment and humans. Used in PV modules, its long biological half-life (estimated at 16–38 years) directly affects the accumulation of this element in the organisms of plants, animals, and humans. Cadmium and its compounds enter the body mainly through the respiratory tract (10–40%). Cadmium inhaled with air, most often as CdO, accumulates in the lungs (10%), and the rest goes into the bloodstream. Much less cadmium enters the body through the digestive tract, about 6%. The penetration of cadmium into the body results in cell damage and poses a serious health risk. For this reason, the recycling processes must be carefully carried out so that the risk to human health and the environment is minimal.

There are several companies in Europe that recycle photovoltaic panels. Veolia Environment SA, in cooperation with PV Cycle, is responsible for the recovery of silicon, glass, and aluminium. Association AISBL is located in France and Relling Glass Recycling Gmbh in Germany. The recycling of thin-film materials is carried out by Lfficiency Holding Gmbh in Germany. In all plants, the end products are glass, aluminium, and high-purity silicon [90].

The environmental benefits and profitability of PV recycling are significant because the recovered materials reduce the cost of producing new PVs, save resources, and reduce waste. However, the costs associated with dismantling, transportation, and specialized recycling technologies, which are currently not economically viable, are still a significant obstacle. However, ongoing technological progress and research efforts in this area can overcome these challenges and increase the profitability of PV recycling.

The European Union, responding to the energy crisis in 2022, accelerated the implementation of photovoltaics, achieving a 50% increase to 28 GW in 2022 [90]. The Green Plan, which sees the decarbonization of the European Union’s energy system as key, sets climate targets for 2030 and 2050. Photovoltaic systems are expected to play a key role in this process. According to the Solar Energy Strategy, Europe plans to obtain 4400 GW of energy by 2030 and 14 TW by 2050 [89].

Similar increases in photovoltaic deployment were realized in 2022 in China to 100 GW, India to 18 GW, and Brazil to 11 GW. In the United States, significant financing for photovoltaics has been implemented, which is expected to contribute to an increase in capacity and the expansion of the supply chain [90].

Solar PV plants are a key technology, but their implementation requires planning for significant land use, environmental protection, social acceptance, and PV recycling management.

Since 2012, recycling of PV modules has been mandatory in the European Union under the Waste Electrical and Electronic Equipment Directive. PV modules are classified as Category 4 “large equipment” for which minimum recovery targets have been set, which must achieve an 85% recovery rate and an 80% preparation for reuse rate. However, there are currently no regulations or benefits for returning PV modules for recycling, resulting in low recycling rates and a high risk of illegal disposal. The biggest problem is the high cost and low profitability of PV recycling.

Currently, the recycling rate of PV modules is not precisely known. The global recycling rate in 2019 was about 14%; it is predicted that by 2030 it could reach 35% and by 2050 70%, assuming a high recycling scenario [89].

The situation is similar with the development of recycling processes for various photovoltaic technologies that are not fully developed. On average, solar modules are divided into (i) glass—54.7%, (ii) aluminium—12.7%, (iii) glue—10%, (iv) silicon—3.1%, and (v) other materials—19.5%. The above percentages may vary depending on the type of photovoltaic module. For a crystalline silicon (c-Si) module, the weight distribution is as follows:

➢ Module area—75%;
➢ Polymer components—10%;
➢ Aluminium—8%;
➢ Silicon material—5%;
➢ Copper—1%;
➢ Other materials—1%, including traces of silver—0.1%.

The advantages of crystalline silicon technology are cost effectiveness, durability, and stability, which translates into an operating period exceeding 25 years.

Thin-film technology modules are mainly composed of glass, polymers, metals, and thin layers of semiconductor materials. The production process of these materials is more cost effective compared to crystalline silicon and is associated with lower energy consumption and a smaller carbon footprint. However, the energy efficiency is 10–20% lower than with silicon modules [90].

Taking the above into consideration, recycling should be effectively managed by implementing appropriate legal regulations and waste management. In this way, the impact on the environment can be minimized and the benefits of recycling PV modules can be maximized.

## 7. SWOT Analysis of Scintillation Material Toxicity

SWOT analysis (presented in Table 3) of scintillation material toxicity allows us to understand both their potential hazards and the opportunities associated with their use.

SWOT analysis of scintillation material toxicity reveals both their strengths and weaknesses, as well as opportunities and threats related to their use. Despite high detection efficiency, the toxicity of some components is a significant problem that requires action to develop safer alternatives and better practices in toxic material management.

### 7.1. Recycling of Scintillators

Scintillator recycling can be divided into several steps, as presented in Table 4.

Spent scintillators, like other radioactive or potentially harmful materials, must be properly managed to minimize the risk to human health and the environment. The process for handling used scintillators depends on the type of material they are made of, their chemical composition, and the degree of potential radiological or toxic hazard. Table 5 lists selected methods for handling used scintillators.

Finally, hygroscopic scintillators such as sodium iodide must be disposed of in special facilities due to their susceptibility to moisture absorption, which leads to degradation of the material. For example, in the case of bismuth germanate (BGO), it is possible to process and recover components such as bismuth or germanium due to its relatively low toxicity.

### 7.2. Impact of Scintillators on CO_2_ Emissions

Scintillator production is a complex technological process that can have a significant impact on the environment, including carbon dioxide emissions. Scintillators are made of various materials, such as crystals, liquids, and plastics, which require appropriate raw materials and advanced production technologies. The theory of scintillator production in terms of CO_2_ emissions is based on the analysis of the technological process and related factors that affect the environment.

First, we proposed to analyse the raw materials and CO_2_ emissions associated with their acquisition. As was described previously, the scintillation materials are produced from various raw materials, depending on the purpose and type of scintillator. The following briefly describes some of these materials:

- Inorganic crystals: The most used materials are sodium iodide (NaI), caesium iodide (CsI), gallium oxide, or gadolinium oxide. The extraction and processing of such raw materials are energy-intensive processes leading to CO_2_ emissions.
- Organic materials (e.g., plastic scintillators): The production of plastic scintillators requires crude oil-based raw materials, which are obtained in refinery processes that generate CO_2_.

The acquisition of inorganic and organic raw materials is therefore associated with emissions resulting from extraction, transport, and processing, which significantly affect the carbon footprint of scintillator production.

Another important aspect involves the synthesis and processing of scintillator materials. Scintillator production involves chemical and physical processes, such as:

- Synthesis of scintillator crystals: This is a highly energy-intensive process, especially for large single crystals, which requires high temperatures (even above 1000 °C) and long growth times. An example is the Czochralski method, which consumes a large amount of electrical energy, which is associated with CO_2_ emissions dependent on the energy source.
- Polymerization for plastic scintillators: The polymerization process (e.g., acrylic or styrene) also requires energy and leads to greenhouse gas emissions from the use of organic compounds and their processing.

Processing of scintillator materials to obtain the appropriate purity and structure, required for the correct operation of the scintillator, also generates CO_2_ due to the need for high temperatures, chemicals, and specialized equipment.

Moreover, recycling and disposal aspects and impact on CO_2_ emissions should be taken into consideration. Some scintillator materials, such as NaI or CsI, are difficult to recycle and require special disposal conditions, which generates additional environmental and emission costs. Plastic scintillators are easier to recycle, but there is still a challenge in large-scale processing. From the perspective of emission reduction theory, implementing recycling procedures that allow the recovery and reprocessing of scintillator materials could significantly reduce the overall CO_2_ emissions associated with their production and consumption.

By understanding the intricacies of recycling and disposal, organizations can mitigate potential environmental impacts associated with scintillators and meet regulatory requirements. Some scintillators, especially those containing heavy metals, can be toxic or hazardous to health and the environment. For example: In NaI(Tl) thallium is a heavy and toxic metal, so scintillators based on it must be used with appropriate precautions. ZnS:Ag can also pose some environmental problems, but in lower concentrations than sodium iodide. Plastic scintillators are generally less toxic but may contain chemicals harmful to health in the production process.

In any case, when using scintillators, it is important to follow safety rules and appropriate regulations regarding hazardous materials to minimize the risk [91,92,93]. Moreover, disposal of scintillators requires special attention through:

- Compliance with regulations: Disposal must comply with local, state, and federal regulations regarding hazardous materials, especially if the scintillator contains any radioactive isotopes or hazardous chemicals;
- Hazardous waste: Scintillators contaminated with radioactive waste are classified as hazardous materials and must be disposed of through a licensed hazardous waste disposal service;
- Landfill: Non-radioactive scintillator waste can be disposed of in landfills, following strict guidelines to prevent environmental contamination;
- Incineration: In some cases, incineration can be a waste reduction method, but it must be handled carefully to avoid the release of harmful emissions;
- Specialty facilities: Some facilities specialize in the disposal of scientific instruments and materials and may have the capabilities to safely handle scintillators.

## 8. Internal and External Determinants in ESG Strategies

All technologies described above should aim to implement the future of green energy in industry, including ESG strategies. However, let us ask ourselves at the end whether this is really the case. Do scientists and entrepreneurs consider environmental protection as a major factor in future work? If so, how far do the boundaries of manufacturing products and devices reach in terms of energy and ecological safety? It is known that completely “clean energy” does not exist—the production of each installation for energy production also requires the use of electricity and is associated with the production of potential waste. Everything must be done to minimize it at each stage of material or device production. The environmental social governance (ESG) perspective should be the main key indicator, not only for industry, but also for the scientists taking into account carbon footprint reduction and ecological and energetic security [94,95]. Currently, the ESG strategy faces many challenges to implement it in companies. Future trends towards the development of ESG strategies should consider all environmental aspects related to the production of a product in line with the principles of green chemistry, i.e., “from cradle to cradle” and not “cradle to grave”. The ESG strategy should become a vital factor in corporate/science competition. For companies to achieve their long-term goals, they should implement and develop ESG strategies also through the use of new technologies or solutions, e.g., artificial intelligence, so that their activities are aimed at sustainable development. It is desirable to use materials obtained in an ecological way, without the use of toxic solvents or inorganic catalysts. It is also advisable to use materials obtained in a one-to-three-stage production process and not multi-stage synthesis, and to use such methods of obtaining products that do not generate harmful by-products. It is also desirable to plan the use of used materials for other purposes towards partial or complete recycling. The ideal solution is industrial symbiosis, where waste from one enterprise becomes a substrate in the production of another enterprise. The ESG strategy is associated with the so-called flower of innovation related to the design of industrial products, taking into account the five aspects important for the final effect, namely: technology and production, financial aspects, social context, design and styling, and human factors [96].

## 9. Conclusions

Currently, the world market of photovoltaics based on crystalline silicon and cadmium tellurate has respective shares of 97% and 3% (as of 2022). From the toxicity point of view, the takeaway message should concentrate on the materials and production of modules and cells, because it brings the highest risk in terms of negative impact on the environment and human health. To take care of the reduction of the impact of already mature technology, the principal focus should be given to recycling processes because they play a key role in improving the sustainability of industrial processes. Looking into future, emerging technologies will most likely take place over outdated technology and will focus on renewable, processable, and toxically neutral components using carbon-neutral processes with high rate of recyclability.

In summary, the world is looking for new renewable sources of energy, among which PVs are becoming more important in solving these climate change issues. The growing awareness of climate change has increased the share of renewable energy sources (RESs) as alternative energy. The greatest challenge is to provide electrical energy from PVs and other RESs when fossil fuel resources are declining, and global warming is increasing. It is believed that PVs, wind, and other renewable energy sources will represent a major portion of the future energy mix.

In order for PV system power generation to be as close as possible to the concept of “zero emission”, it is necessary to consider ways to reduce CO_2_ emissions during the production phase of PV systems to a minimum level that is justified technologically rather than financially. The following are the areas where the greatest reductions in CO_2_ emissions are expected:

- Increasing the production of PV modules in the EU, which is estimated to save 40% of CO_2_ emissions compared to modules imported from China;
- Increasing the efficiency of photovoltaic modules;
- Increasing the lifetime of photovoltaic panels;
- Eliminating aluminium from production by developing frameless glass-to-glass module technology;
- Increasing the share of electricity from renewable energy sources in the production phase of PV systems;
- Conducting research on new PV technologies for applications in areas where conventional silicon PV cells are not cost-effective, such as low-light environments, greenhouse roofs, etc.;
- Conducting research and deployment of binary technologies such as combining PV panels with solar panels;
- Conducting research on technologies for recycling PV panel components and producing new PV panels from them.

Finally, we would like to mention low-emission alternatives and production optimization processes. In terms of both theoretical and practical CO_2_ reduction, key actions include:

- Using low-emission energy sources in production, such as solar or nuclear energy, especially for high-temperature synthesis;
- New synthesis methods: developing production methods that operate at lower temperatures or use alternative technologies, such as microwave crystal growth, which reduce the total energy required to produce scintillators and solar cells;
- Replacing inorganic materials with organic materials where possible—organic materials, although less efficient in detecting high-energy radiation, may offer lower CO_2_ emissions during production.

From a scientific point of view, scintillator and solar cells production can be optimized in terms of CO_2_ emissions by:

- Minimizing energy consumption in the synthesis and processing of materials;
- Using renewable or easier to recycle raw materials and materials;
- Improving production technologies with an emphasis on energy efficiency.

This theory emphasizes the need to search for new production methods and implement more ecological practices to reduce the carbon footprint and environmental impact during scintillator and solar cell production.

## Figures and Tables

**Table 1:** A list of the most frequently used dopants for scintillators and the functions they perform.

Scintillator	Dopant/s	Function	Source
Caesium iodide (CsI(Tl))	Thallium (Tl)	Improves the ability to detect gamma radiation and increases the efficiency of converting radiation energy into light. NaI(Tl) is one of the most commonly used scintillators in nuclear medicine and radiation detection.	[18,19]
Sodium iodide (NaI(Tl))			
Lutetium oxide (Lu_2_O_3_)	Cerium (Ce)	Improves the decay time (speed of light emission) and increases detection sensitivity, especially to gamma radiation. Cerium is used in scintillators used in positron emission tomography (PET) and high energy detection.	[20]
Gadolinite (Gd_2_O_2_S)			[21]
LSO (Lu_2_SiO_5_)			[22]
Zinc sulphide (ZnS)	Europium (Eu)	Increases emission brightness and is used in alpha and low-energy radiation detectors. Europium-doped scintillators are used in X-ray monitors and screens.	[23]
	Samarium (Sm)	Dopant enables efficient detection of alpha radiation and neutrons. ZnS is used in the detection of alpha particles and thermal neutrons.	[24]
Garnets (Y_3_Al_5_O_12_, YAG)	Yttrium (Y)	Dopant is used to increase sensitivity to X-rays and gamma rays and improve mechanical properties and thermal stability of scintillators. YAG is used in medical imaging systems.	[25]
Gadolinite (Gd_2_O_2_S)			[26]
Lithium fluoride (LiYF_4_)	Praseodymium (Pr)	Improves decay time and light efficiency, especially in scintillators used for detecting neutron radiation.	[27]
Gadolinite (Gd_2_O_2_S)	Terbium (Tb)	Dopant enables intense light emission due to absorption of X-rays. These materials are used in X-ray screens and beta radiation detection.	[28]
Garnets (Gd_3_Ga_5_O_12_)	Neodymium (Nd)	Improves decay time and is used in gamma and X-ray detection, especially in complex medical and imaging systems.	[29]
Fluorides (LiHoF_4_)	Holmium (Ho)	Is used to increase the sensitivity of scintillators to neutrons and is used in thermal neutron detection systems.	[30]
LSO (Lu_2_SiO_5_)	Lutetium (Lu)	Increases the density of the scintillator material, which improves the detection efficiency of high-energy radiation such as gamma radiation. Lutetium scintillators are mainly used in PET systems and ionizing radiation detection.	[31]

**Table 2:** List of factors affecting the durability and lifespan of scintillators.

Factor	Effects	Source
Exposure to radiation	Radiation dose: Long-term exposure to radiation can lead to degradation of scintillators, especially in the case of organic materials. High doses of radiation can cause damage to the crystal structure of the scintillator, leading to a reduction in its light output. Radiation damage: Some scintillators, especially those based on organic materials (e.g., polystyrene or polyvinyltoluene), are susceptible to radiation damage. This damage can manifest itself in colour changes, reduced light output, and reduced operating life.	[59,60,61,62]
Moisture	Hygroscopicity: Some scintillators, such as sodium iodide (NaI), are hygroscopic, meaning they absorb moisture from the environment. Moisture can cause degradation of the material, leading to a loss of optical efficiency and a reduction in the transparency of the scintillator. Housing tightness: To prevent degradation caused by moisture, hygroscopic scintillators must be stored in tight, airtight housings, often filled with inert gases such as argon, or in moisture-proof shields.	[14,17,63]
Thermal factors	High temperature: High temperatures can damage scintillators, especially organic materials, resulting in degradation of their molecular structure. In extreme cases, this can cause cracking, colour changes, and loss of scintillation ability. Thermal phenomena: Sudden changes in temperature can lead to thermal stresses in scintillators, especially in crystalline materials such as bismuth germanate (BGO) or sodium iodide (NaI). These stresses can cause cracks or other mechanical damage.	[17]
Material aging	Chemical changes: Over time, some scintillators may undergo chemical aging processes that affect their performance. This can include oxidation, molecular degradation, or reactions with environmental contaminants. Mechanical changes: As a result of aging, the mechanical properties of a scintillator, such as brittleness or shock resistance, can deteriorate. This can affect their ability to operate for long periods in harsh environments.	[63,66]
Impurities and crystal defects	Contamination during manufacturing: The presence of impurities in the scintillator material can affect its service life. These impurities can reduce the efficiency of the conversion of radiation to light and accelerate the degradation of the material. Crystal defects: In the case of inorganic scintillators such as BGO, LSO (lutetium silicate), or NaI, defects in the crystal structure can affect the scintillator properties and lead to degradation of the optical performance over time.	[14]
Mechanical factors	Vibration and shock: Crystal scintillators such as NaI(Tl) or BGO are susceptible to mechanical damage. Vibrations, shocks, or drops can cause cracks, which in turn reduces their performance. Mechanical stress: Under conditions of strong mechanical stress, scintillators can deform, which negatively affects their performance.	[65]
Exposure to light	UV light exposure: Some scintillators, especially organic ones (e.g., PVT—polyvinyl toluene), are sensitive to UV radiation, which can lead to material degradation. Prolonged exposure to sunlight can reduce their ability to emit light after absorbing ionizing radiation.	[66,67]
Proper maintenance and storage	Dry storage: Hygroscopic scintillators, such as NaI(Tl), require proper storage in airtight containers in a dry environment. Temperature and humidity control: For many scintillators, especially those sensitive to moisture and temperature changes, it is crucial to control the environment in which they are stored and used.	[14,68]

**Table 3:** SWOT analysis of scintillation material toxicity.

Strengths	Weaknesses	Opportunities	Threats
Efficiency in radiation detection:	Toxicity of some components:	Development of alternative materials:	Technological competition is growing:
Scintillation materials, despite potential toxic effects, offer high efficiency in radiation detection, which is crucial in many fields such as medicine, industry, and science.	Some scintillators, such as NaI(Tl) containing thallium, are toxic and may pose a risk to human health and the environment.	There is potential to develop new, less toxic scintillators based on alternative chemical components.	Other radiation detection technologies, such as semiconductor detectors, may become more popular, which could limit the use of scintillators.
Possibility of chemical modification:	Chemical Exposure:	Increased demand for radiation detection:	Environmental Regulations:
Scintillators can be modified by adding dopants, which allows for improving their efficiency and reducing the toxicity of some components.	Scintillator manufacturing and use processes often involve exposure to toxic chemicals, which poses a health risk to workers and users.	Increased demand for radiation detection technologies in medicine and industry creates opportunities for innovation and improved toxicity practices.	Increased regulations regarding toxic chemicals may impact scintillator production and introduce additional restrictions.
Diversity of applications:	Recycling Issues:	Safety regulations and standards:	Negative health effects:
Due to their efficiency, scintillators are used in a wide range of applications, which can increase their value and importance in industry.	The high toxicity of some materials can limit their recycling capabilities, leading to waste management issues.	Introducing stricter regulations regarding toxic substances can lead to improved manufacturing practices and reduced risk.	Increasing public awareness of the toxicity of some materials may lead to a decrease in acceptance of scintillators containing hazardous substances.

**Table 4:** Recycling processes in scintillators.

Type of Process	Description
Identification and segregation	The used scintillators are assessed to identify their chemical composition and potential radioactive contamination. In the event that the scintillator is radioactive, it must be properly disposed of as radioactive waste. If it shows no contamination, the materials can be recycled.
Dismantling and material recovery	Scintillators consist of various components that can be recovered: Scintillator crystals: Crystals such as NaI(Tl) (sodium iodide activated by thallium) or LSO (lutetium oxyorthosilicate) are valuable because of the rare metals (e.g., lutetium) that are hard to obtain. These crystals can be reused in the production process of new scintillators after appropriate chemical treatment to remove impurities. Heavy metals and rare elements: Toxic and rare elements such as thallium, lutetium, and gadolinium can be recovered from scintillators. The recovery process involves dissolving the materials in suitable solvents and separating the valuable elements using techniques such as solvent extraction or distillation. Plastics: In the case of organic (plastic-based) scintillators, the material can be recycled or disposed of, depending on its technical condition and value. Plastic scintillators can be mechanically recycled, but their use after recycling may be limited.
Processing and reuse	Materials recovered from recycling can be reused to produce new scintillators, electronic components, or in other industrial processes. Recovered rare elements such as lutetium are particularly valuable in the technology industry and can be used not only in radiation detectors but also in other electronic devices.
Waste and disposal	Residues from the recycling process that cannot be reused must be properly disposed of. In the case of radioactive materials, strict regulations must be followed regarding the storage of radioactive waste. Chemical materials such as contaminated plastics are subject to special disposal procedures in accordance with environmental regulations.
Process safety	Scintillator recycling requires appropriate safety measures, both due to the possibility of exposure to radiation and contact with toxic chemicals such as thallium or heavy metals. Workers must be adequately protected and processes must be carried out in controlled conditions to minimise risks.

**Table 5:** Selected methods of dealing with used scintillators.

The Way to Proceed	Procedure Description
Hazardous waste disposal	For scintillators that contain toxic or radioactive materials, disposal is carried out in accordance with hazardous waste regulations. These materials must be collected, transported, and processed in accordance with stringent safety standards. Radioactive scintillators, which may contain radioactive elements, must be processed in special facilities adapted to the treatment of radioactive waste. This waste is typically stored in safe locations, such as underground radioactive waste storage facilities.
Recycling of materials	Many scintillators contain valuable elements such as lutetium (Lu), bismuth (Bi), and caesium (Cs) that can be recycled and reused. The recycling process involves the recovery of valuable materials that can be used to produce new scintillators. Sodium iodide (NaI) or caesium iodide (CsI) can be purified after use, where impurities are removed and the material is reused.
Mechanical and chemical processing	Some scintillators can be subjected to mechanical (e.g., grinding, separation) and chemical (e.g., dissolving in appropriate chemicals to separate components) processes that allow materials to be recovered for reuse or waste to be prepared for safe disposal. For example, bismuth germanate (Bi₄Ge₃O₁₂) can be processed in such a way as to recover bismuth or germanium for further industrial use.
Secure storage	If scintillators are not recyclable or reusable, they are properly disposed of in safe conditions. In the case of inorganic scintillators, which are not radioactive, they can be treated as industrial waste that is disposed of in landfills adapted to hazardous waste. Organic scintillators, such as polystyrene (PS) or polyvinyltoluene (PVT), can be incinerated in specialised installations, provided that the emission standards are met.
Disposal of radioactive materials	Scintillators containing radioactive materials, such as lutetite-177 or other isotopes used in certain types of scintillators, must be treated as radioactive waste. At the end of their useful life, they are stored in facilities intended for the storage of radioactive waste of low or medium activity. Radioactive waste can also undergo decontamination processes, which allow its radioactive activity to be reduced before further processing.
Recovery	In some cases, scintillator remanufacturing is possible, in which older or worn materials are refurbished and returned for reuse. This process may include repairing mechanical damage or cleaning the material to restore its original scintillation properties.
Regulatory Rules and Standards	The handling of used scintillators is strictly regulated by national and international regulations on environmental protection and hazardous waste management. Regulatory standards depend on the type of material and the country in which the scintillators are operated. Organizations such as the International Atomic Energy Agency (IAEA) or national environmental protection agencies (e.g., the National Atomic Energy Agency in Poland) regulate regulations on the safe handling of radioactive and hazardous waste.

## Data Availability

The authors declare that the data supporting the findings of this study are available within the paper.
